# A pilot study to assess the safety and usefulness of combined transurethral endoscopic mucosal resection and en-bloc resection for non-muscle invasive bladder cancer

**DOI:** 10.1186/s12894-019-0486-0

**Published:** 2019-06-24

**Authors:** Yasushi Hayashida, Yasuyoshi Miyata, Tomohiro Matsuo, Kojiro Ohba, Hideki Sakai, Mitsuru Taba, Shinji Naito, Keisuke Taniguchi

**Affiliations:** 1 Department of Urology, National Hospital Organization Ureshino Medical Center, 2436 Shimosyuku, Ureshino, 843-0393 Japan; 2 Department of Urology, Nagasaki University Graduate School of Biomedical Sciences, 1-7-1 Sakamoto, Nagasaki, 852-8501 Japan; 3 Department of Pathology, National Hospital Organization Ureshino Medical Center, 2436 Shimosyuku, Ureshino, 843-0393 Japan

**Keywords:** Endoscopic mucosal resection, En-bloc resection, Non-muscle invasive bladder cancer, Safety, Outcome

## Abstract

**Background:**

Transurethral resection (TUR) is the standard operation used for non-muscle invasive bladder cancer (NMIBC). Although most solid tumors are principally removed via single block resection without incising the mass, disruption of the lesion is unavoidable in traditional TUR. Furthermore, pathological diagnosis is often difficult due to heat-related denaturation of tissues in TUR. Although transurethral en-bloc resection is useful for judging tumor invasion, it is associated with a prolonged operative duration. We attempted to show the safety and usefulness of combined endoscopic mucosal resection (EMR) and en-bloc resection in NMIBC patients.

**Methods:**

We investigated 39 patients with clinical NMIBC who were treated using our original EMR + en-bloc resection technique, which involved removal of the tumor mass that protruded from the mucosa, using a polypectomy snare similar to that used for EMR. The residual lesion was removed using en-bloc resection. The operative period, duration of hospitalization, and recurrence rates were compared with those of conventional TUR (*n* = 31).

**Results:**

The mean (standard deviation, range) time interval for EMR and total operative duration were 1.6 (1.1, 1–5) min and 18.3 (10.5, 3–48) min, respectively. The total operative duration was comparable to that of TUR (17.3 min, *p* = 0.691). The mean duration of catheterization in the EMR + en-bloc resection group (4.2 days) was also similar to that in the TUR group (3.7 days; *p* = 0.285). No severe complications were observed with EMR + en-bloc resection. The pathologists were able to determine tumor invasiveness with considerable certainty in all specimens obtained via the EMR + en-bloc procedure than via TUR, and the difference in the ease of diagnosis was statistically significant (*p* = 0.016). Recurrence rates were comparable (*p* = 0.662) between the EMR + en-bloc (15.4%) and TUR groups (19.4%).

**Conclusions:**

Our results demonstrated that the EMR + en-bloc resection technique is feasible, safe, and useful for treating patients with NMIBC. Furthermore, this technique helps provide a more accurate pathological diagnosis.

## Background

Bladder cancer (BC) is one of the most common urological malignancies in men [1]. Approximately 75–85% of newly diagnosed malignancies that are limited to the bladder mucosa or submucosa are classified as non-muscle invasive bladder cancers (NMIBCs) [2]. Transurethral resection (TUR) remains the gold standard for the treatment of NMIBC. The choice of a radical resection procedure is an important determinant of the outcome in patients with NMIBC. In addition, reaching an accurate diagnosis, especially in the pathologic stage (pT), is important to choose appropriate treatment strategies in these patients. Furthermore, an accurate histopathological diagnosis leads to reduction of overall treatment costs, because an unnecessary second TUR procedure or adjuvant intra-vesical therapy is avoided. Thus, the goal of TUR in early BC is to completely excise visible masses and obtain tissues for an accurate pathological diagnosis of the tumor.

Although TUR is an established and traditional treatment approach, it has various disadvantages. An accurate pathological diagnosis is often difficult because the tumor is removed piecemeal, and the extracted specimens often show morphological changes due to heat denaturation and tissue shrinkage caused by high energy of the resection loop [3, 4]. In addition, some specimens are rendered inadequate due to disorientation and absence of the detrusor muscle tissue. Moreover, although progression rate of NMIBC is relatively low, some researchers are of the opinion that the high number of exfoliated and scattered cancer cells produced during TUR, could lead to metastasis and recurrence due to subsequent seeding and re-implantation [5].

The oncological principle for almost all solid cancers the removal of the tumor via single block resection without incising and cutting into the mass. However, disturbing the tumor mass is unavoidable in traditional TUR. To solve these problems, an ‘en-bloc’ resection technique was suggested ~ 20 years ago [6]. Currently, there is a general agreement that en-bloc resection of bladder tumors is useful and safe for treating patients with NMIBC [7]. However, an international guideline on NMIBC recommends that only small-sized tumors (defined as those with a diameter < 1 cm) can be resected en-bloc [8]. In addition, en-bloc resection may require a prolonged operative duration, and an appropriate line of resection can be missed due to bleeding associated with large-sized tumors. Thus, en-bloc resection is not usually employed in patients with relatively large tumors.

Endoscopic mucosal resection (EMR) is a well-defined technique used for the operative removal of gastrointestinal masses. EMR has been used to extract precancerous polyps, early-stage malignant lesions in the esophagus and colon, and gastric cancer lesions. Recently, EMR has been reportedly utilized to remove large lesions in the gastrointestinal tract [9].

Therefore, we paid special attention to EMR for the operative treatment of BCs, especially the large-sized tumors. We hypothesized that performing EMR for the raised portion of a BC lesion can shorten the operative time required for subsequent en-bloc resection in patients with NMIBCs. To test this hypothesis, we investigated the operative time period and duration of both catheterization and hospitalization required, while using the EMR + en-bloc resection technique to treat patients with NMIBCs. In addition, we compared the measures of these variables to those associated with a conventional TUR procedure.

## Methods

### Patients

We received approval for the protocol (including the indication for patient selection), from the institutional review board of the National Hospital Organization Ureshino Medical Center to perform the EMR + en-bloc resection technique in selected patients, and to evaluate occurrence of complications, patient outcomes, and pathological diagnoses in those who were operated using either the combination technique or TUR. Written informed consent was obtained from all patients included in the study. In our hospital, while conventional TUR is performed as a standard procedure for the removal of all tumours diagnosed as NMIBC, en-bloc resection (without EMR) is utilized in patients with ≤3 lesions, each with diameter < 1.5 cm. In this study, we hypothesized that EMR may help reduce the operative duration required for the removal of relatively larger tumours. Therefore, we decided to include bladder tumour diameter ≥ 1.5 cm as the indication for our EMR + en-bloc method. The institutional review board permitted the employment of EMR + en-bloc operative method in patients with ≤3 tumours of diameters ≥1.5 cm, with prior detailed informed consent taken from the concerned patients. Therefore, we provided patients meeting the selection criteria, with in-depth information regarding both EMR + en-bloc and conventional TUR techniques including the surgical method, advantages, predictable complications, and cost of each technique. The combined surgical approach was finally chosen after exhaustive consultations with each selected patient and family, in accordance with the rules established by the institutional review board. Consequently, 39 patients finally underwent the EMR + en-bloc resection between January 2013 and December 2017.

However, for a comparative study of the clinicopathological features, we retrospectively collected and analyzed data of those bladder cancer patients, who underwent TUR and had > 3 tumors, with diameters < 1.5 cm or > 6 cm (the diameter of the largest mass operated using the combination technique). Finally, we included data of 80 patients, who underwent TUR for NMIBC without metastasis (diagnosed clinically), in this study. Those who received neo-adjuvant chemo- and radiotherapy were excluded. The baseline clinicopathological features of these patients at the time of the operation are shown in Table 1. Although this study is not a randomized clinical trial, we found no significant differences in the clinicopathological characteristics between the participants included in the EMR + en-bloc and the TUR groups (Table 1). Patients included in both groups received prophylactic antibiotics (e.g., cephalosporins) pre- and postoperatively.

**Table 1:** Clinicopathological features at operation

	EMR + en bloc	TUR	*P* value
	*n* = 39	*n* = 31	
Gender			0.591
Male	24 (61.5)	21 (67.7)	
Female	15 (38.5)	10 (32.3)	
Tumour			0.148
Primary	36 (92.3)	25 (80.6)	
Recurrent	3 (7.7)	6 (19.4)	
Location of main tumour			0.879
Lateral	14 (35.9)	13 (41.9)	
Posterior	13 (33.3)	9 (29.0)	
Dome	7 (17.9)	4 (12.9)	
Trigone	5 (12.8)	5 (16.1)	
Pathological grade			0.896
Grade 1	12 (30.8)	10 (32.3)	
Grade 2	19 (48.7)	16 (51.6)	
Grade 3	8 (20.5)	5 (16.1)	
Pathological T stage			0.915
Ta	19 (48.7)	16 (51.6)	
T1	18 (46.2)	14 (45.2)	
T2	2 (5.1)	1 (3.2)	

*EMR endoscopic mucosal resection, TUR transurethral resection*

### Surgical technique

**Fig. 1:**
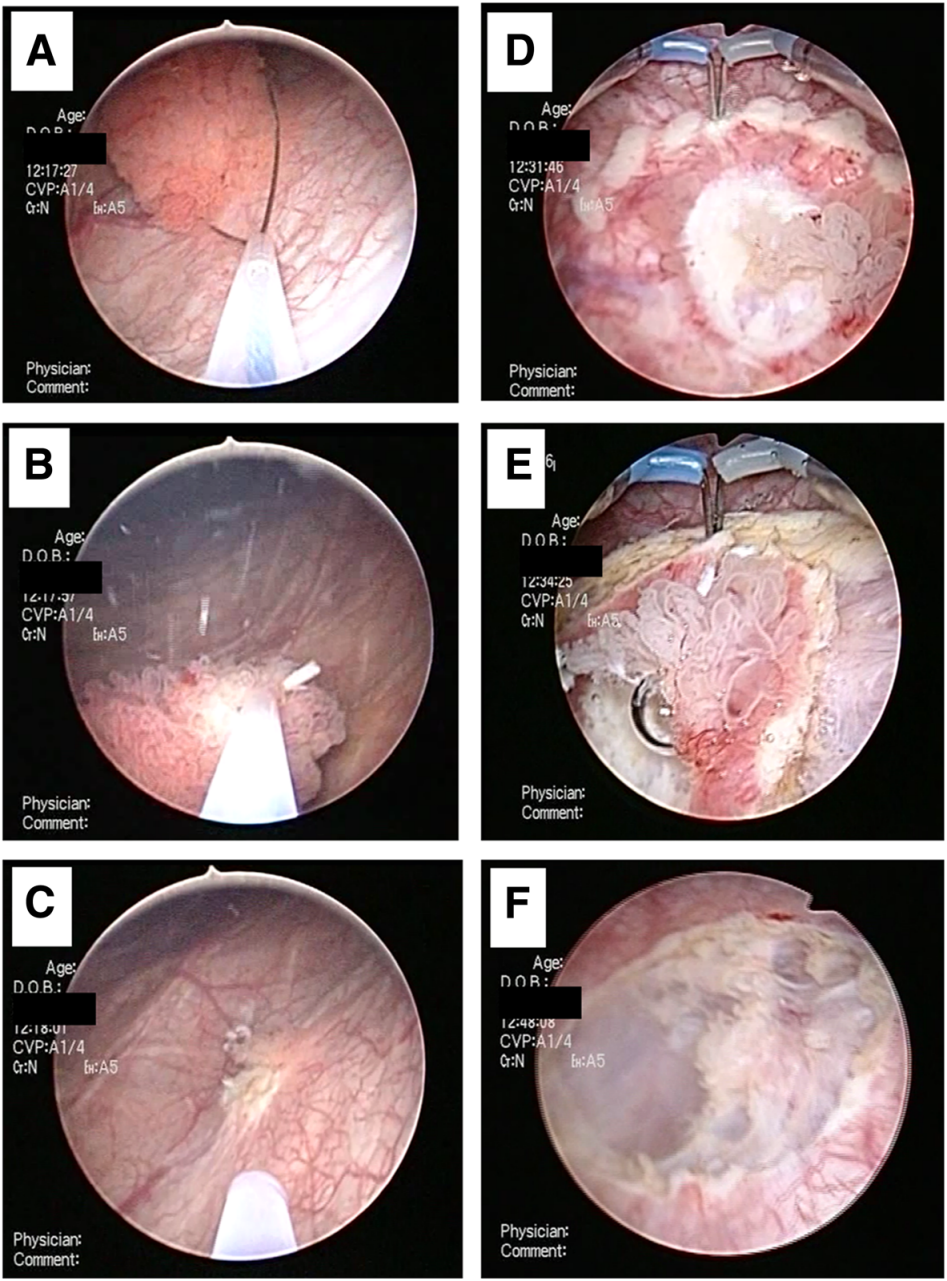
A description of the surgical technique for performing EMR + en-bloc resection. **a** A snare is inserted at the base of the pedunculated tumor; **b** the snare is placed close to the bottom of the tumor, and EMR is performed; **c** a flat or residual tumor mass is shown; **d** a circular incision is created around the residual tumor, maintaining a distance of approximately 5–10 mm from the tumor edge; **e** en-bloc resection is performed, and **f** the tumor is completely resected. (EMR: endoscopic mucosal resection)

Sequential images describing the EMR + en-bloc resection are shown in Fig. 1. Firstly, a section of the target tumor mass protruding from the mucosa, was incised using a polypectomy snare (CAPTIVATOR II, Boston Scientific, Marlborough, MA, USA), similar to the one used in EMR [10]. Along with the polypectomy snare, we also used the monopolar ERBE VIO 300D (Endo Cut Effect 2, Tubingen, Germany) electrosurgical device to perform EMR. However, in this step, our method differed from that used for conventional EMR because, we did not inject a fluid or a gel into the submucosal layer, since pooling of the injected substance in the submucosal region is difficult due to the anatomical characteristics of the bladder wall [11]. If the tumor was too large to be resected with a single application of the snare, it was used once more to flatten the residual tumor or mucosal tissue. Subsequently, a circular incision was created around the residual lesion using a T-shaped electrode needle TUR-in-saline system (Olympus®, Tokyo, Japan), while maintaining a distance of approximately 5–10 mm from the tumor edge, for subsequent en-bloc resection, similar to the technique employed for endoscopic submucosal dissection of superficial gastrointestinal tumors [12].

In the control group, conventional TUR was performed in 31 NMIBC patients, who were matched with those in the study group, based on factors including tumor diameter and clinical stage. An intravesical instillation of anthracycline antibiotics was performed in these patients in the immediate postoperative period, to prevent the tumor from spreading. Postoperative complications were assessed using the Clavien-Dindo classification [13]. Furthermore, two pathologists (MT and SN) determined the histopathological diagnoses (in all patients), which were used to prognosticate future outcomes. The pathological diagnosis made by each of the 2 observers were the same for each patient.

In this study, all operations were performed by a single surgeon (KT). He had experience in operating over 700 and 30 patients using TUR and en-bloc resection (for small masses) techniques, respectively. However, he had no special training in EMR. Therefore, he was verbally guided by a surgeon experienced in colonic EMR during the operative procedures for the first 3 patients.

### Follow-up and outcomes

After TUR, we investigated all patients’ cystoscopy and cytology results once every three months for 2 years and then every 3–6 months for 5 years depending on the pathological features. The mean (range) follow-up period of the study population was 26 (9–60) months.

### Statistical analyses

The Mann-Whitney U test was used for comparisons involving continuous variables because of the relatively small number of patients. The chi-square test and Fisher’s exact test were used to compare categorical data. All statistical analyses were two-sided and performed using StatView for Windows (version 5.0; Abacus Concepts, Inc., Berkeley, CA, USA) software. *P* values < 0.05 were considered representative of statistical significance.

## Results

The tumors in all patients were successfully removed using en-bloc resection, and all extracted specimens were found to include detrusor muscle tissue. The information on the operative procedures is shown in Table 2. There were no significant differences in the patients operated using either method, with respect to factors including age at the time of operation, number of tumors, or size of the main lesion. The mean (standard deviation [SD], range) operative periods for EMR and en-bloc resection were 1.6 (1.1, 1–5) min and 16.9 (10.5, 2–43) min, respectively. The total operative duration for the EMR + en-bloc resection and the TUR groups was 18.3 min (10.5, 3–48) and 17.3 min (9.5, 2–31), respectively, which was not significantly different between the two groups (*p* = 0.691). The operative time periods needed for the EMR + en-bloc resection of tumors with diameters ≥3.5 cm (28.3%; 11/39 patients) are shown in Table 3. While the total operative time for the removal of a 5.5-cm sized tumor was 41 min, the time interval required for EMR was only 2 min. Thus, EMR was completed within 3 min even for relatively large tumors.

**Table 2:** Information on operation and hospitalization after operation

	EMR + En-bloc			Transurethral resection			*P* value
	Mean	SD	Range	Mean	SD	Range	
Age; years	69.7	7.7	55–86	70.5	6.6	53–80	0.635
Tumor number	1.3	0.6	1–3	1.4	0.6	1–3	0.458
Tumor size; cm	2.9	0.8	1.5–5.5	2.6	0.9	1.5–5.0	0.120
Operation time; min							
For EMR	1.6	1.1	1–5	–	–	–	
For en bloc	16.9	10.5	2–43	–	–	–	
Total	18.3	10.5	3–48	17.3	9.5	2–31	0.691
Catheterization: days	4.2	2.3	3–14	3.7	1.4	3–10	0.285
Clavien-Dindo score^a^	*N*	%		*N*	%		0.881
1	7	17.9		6	19.4		

^a^Overall postoperative complications postoperative day 0–90. EMR: endoscopic mucosal resection

**Table 3:** Operation time in tumor ≥3.5 cm

Local of tumour	Tumour size; cm	Operation time; min		
		EMR	En-bloc	Total
EMR + en-bloc				
Lateral	3.5	1	21	22
Dome	4.0	1	21	22
Posterior	3.5	2	42	44
Lateral	4.0	3	10	13
Trigone	5.5	2	39	41
Dome	4.0	2	21	23
Lateral	3.5	1	16	17
Lateral	3.5	1	10	11
Dome	3.5	2	30	32
Lateral	3.5	1	9	10
Dome	4.0	2	5	7
TUR				
Dome	3.5	–	–	19
Lateral	4.5	–	–	39
Lateral	3.5	–	–	28
Posterior	5.0	–	–	31
Lateral	3.5	–	–	23
Lateral	3.5	–	–	20
Lateral	4.0	–	–	21
*P* value	0.826	–	–	0.470

*EMR endosopic mucosal resection, TUR transurethral resection*

Although the surgeon had no prior experience in EMR, he was able to perform it easily from the first operation, with only verbal guidance. In fact, the median time interval calculated for all EMRs in the 1st–5th, 6th–15th, 16th–30th, 31st–35th, and 36th–39th operated patients, was 2 min. We also show comparative data of the TUR group in Table 3. In the TUR group, 7 patients (22.5%) had tumors with diameters ≥3.5 cm, and there were no significant differences in the total operative time periods (*p* = 0.470) required for these patients.

With regard to safety assessment, no severe complications, e.g., acute bleeding, occurred during or after the operation in both the EMR + en-bloc and the TUR groups. None of the patients required a blood transfusion. In the EMR + en-bloc resection group, no patient required conversion to conventional TUR. Although a minor perforation (visible fat tissue) occurred in one patient in the EMR + en-bloc resection group, surgical treatment or peritoneal drainage was unnecessary. Total 7 (17.9%) and 6 patients (19.4%) in the EMR + en-bloc and TUR groups, respectively, experienced grade 1 complications as per the Clavien-Dindo scale. However, no patient had ≥grade 2 complications. The risk of occurrence of complications was found to be similar across both groups (*p* = 0.881, Table 2). The mean duration of urinary catheterization in the EMR + en-bloc resection group (mean, 4.2 days; SD, 2.3 days) was also similar to that in the TUR group (mean, 3.7 days; SD, 1.4 days; *p* = 0.285).

The pathologists were able to determine the invasive status with considerable certainty in all specimens of patients in the EMR + en-bloc resection group. However, both pathologists commented that determining malignant invasion into the bladder submucosal connective tissues was difficult in 6 of the 31 specimens (19.4%) of the patients in the TUR group, due to heat denaturation. Statistical analysis showed that this difference in ease of diagnosis between both groups was statistically significant (*p* = 0.016). These 6 patients further underwent a second TUR procedure because their tumors were judged as high grade, with or without pT1 (invasion of lamina propria) disease, though residual cancer cells were not detected in such specimens.

After a mean follow-up of 12 months, 6/39 (15.4%) and 6/31 (19.4%) patients in the EMR + en-bloc resection and the TUR groups, respectively, experienced recurrence of the bladder mucosal cancer. Thus, the recurrence rate of NMIBCs was found to be similar across both groups (*p* = 0.662).

## Discussion

We demonstrated that the novel EMR + en-bloc resection technique is safe and useful and enables an accurate pathological diagnosis in BCs, though the operative time period and duration of hospitalization required are similar to those observed with conventional TUR. While en-bloc resection has various advantages with respect to diagnosis and treatment of BCs, as compared to those achieved with conventional TUR, it also has its disadvantages, e.g., prolonged surgical duration. Our novel combined approach aimed to solve this main disadvantage of en-bloc resection.

There are various opinions on the suitable tumor size and number of lesions that indicate the need for en-bloc resection. Hurle et al. suggested that patients with a single tumor with diameter < 30 mm and/or those with < 4 lesions are eligible for en-bloc resection [14]. In another study, tumors > 40 mm in diameter were excluded [15]. A tumor diameter > 25 mm has been suggested as a clear contraindication for en-bloc resection [16]. The European Association of Urology guidelines mentioned that small tumors (defined as those with a diameter < 10 mm) can be resected en-bloc [8]. Reports within the past 5 years have shown that the mean diameter of tumors operated using en-bloc resection, was between 1.58–2.63 cm, and the operative duration was between 21.46–58.2 min (Table 4). However, the mean tumor size and operative period for our EMR + en-bloc resection technique were 2.90 mm and 20.0 min, respectively. Based on these results, we suggest that this method can be used to resect tumors more efficiently, as compared with previous en-bloc resection techniques.

**Table 4:** A review of literature on en-bloc resection within recent 5 years

Author: device	*N*	Tumour size; cm	Tumour number	Operation time; min	Reference/Year
E-ERBT					
Kramer: Monopolar	91	2.13 (0.71)	1.48 (0.74)	27.19 (11.96)	[17]/2015
Kramer: Bipolar	65	2.25 (0.71)	1.62 (0.86)		
Hurle	74	1.98 (0.59)^a^	1 (1–4)	–	[14]/2016
L-ERBT					
Liu: Thulium YAG	64	1.31 (0.23)	2.8 (1.2)	48.2 (15.8)	[18]/2013
He: Green-light KTP	45	1.8 (0.8–3.0)	–	21 (12–38)	[19]/2014
Chen: Thulium YAG	71	2.6 (1.4)	1.8 (1.5)	56.5 (37.4)	[20]/2015
Muto: Thulium YAG	55	2.36 (1.47)		33 (14)	[21]/2015
Kramer; Holmium YAG	50	2.63 (0.79)	1.36 (0.56)	29.65 (12.46)	[17]/2015
Kramer: Thulium YAG	15	1.66 (0.73)	2.60 (0.73)		
Migliari: Thulium	58	2.5 (0.5–4.5)	–	25 (12–30)	[22]/2015
Chen: Green-light LBO	83	1.85 (1.07)	1.76 (0.81)	21.46 (10.42)	[23]/2016
Zhang: Vela	38	2.1 (0.8–3.0)	–	23 (15–43)	[24]/2017
D’souza: Holmium YAG	27	1.58 (0.31)	2.5 (1.5)	58.2 (15.8)	[25]/2017

Data were showed as mean (SD or range)

^a^Among 74 patients, 6 underwent a combination of ERBT and TURBT *E-ERBT* electrical en-bloc resection of bladder tumor, *L-ERBT* laser en-bloc resection of bladder tumor, *YAG* Yttrium Aluminum Garnet, *KTP* potassium-titanyl-phosphate, *LBO* lithium triborate

Electrical and laser devices have been mainly utilized for en-bloc resection in BCs. After Saito described the utility and safety of laser en-bloc resection of bladder tumors in 2001 [26], other reports have indicated the effectiveness of Ho:YAG or Tm:YAG laser treatment [17–22, 25]. The advantages of en-bloc laser resection include absence of the obturator reflex, minimal intraoperative bleeding, reduced hospitalization period, and lower complications, as compared to conventional TUR [21, 27, 28]. However, laser resection is inferior to electrical resection in terms of availability and medical economics, because, not every hospital has access to laser devices, which also render the treatment expensive. We emphasize that our EMR + en-bloc resection method has a relatively low cost and can be used commonly, as it does not require a special device. Therefore, our EMR + en-bloc method can be employed worldwide, even in developing countries.

A metanalysis showed that en-bloc resection can provide high-quality specimens for the pathological diagnosis of BC [29]. Our study supports this finding because the extracted specimens of the EMR + en-bloc resection group were clearly suitable for the pathological diagnosis. The histopathological diagnosis is one of the strongest determinants of treatment alternatives in further management of BCs, e.g., second TUR procedure or intra-vesical therapy. It is also used to determine the post-treatment follow-up schedule. An accurate pathological diagnosis leads to suppression of the overall treatment costs and reduces the mental and physical burden on the affected patient. The novel EMR + en-bloc resection approach can therefore be included, while planning an optimal strategy for the treatment and observation of patients with NMIBC.

To perform en-bloc resection of large, malignant bladder tumors, several investigators have used various modified methods and employed new devices. Naselli et al. retrieved tumors with diameters ≤45 mm using Collins loop and laparoscopic forceps [30], while Frische et al. reported performing en-bloc resection of tumors ≤75 mm in diameter, using a water jet dissector and needle knife for transurethral dissection [31]. Unfortunately, up to 45 min were needed for tumor resection in the former procedure, while the authors did not describe the precise operative duration for the latter method. Furthermore, one study evaluated the combined use of electrical en-bloc resection of the tumor (E-EBRT) and TUR to treat patients with NMIBC [13]. In that study, although E-EBRT was performed for single tumor masses ≤3 cm and for those BCs with ≤4 lesions, the en-bloc resection was limited to tumors with ≤3 lesions, and those with diameters ≥4 cm were removed via TUR. However, as shown in Table 3, our EMR + en-bloc resection technique was useful for extracting tumors with diameters ≥3.5 cm. Furthermore, with regard to safety and adverse events, no patient operated using our combined approach required conversion to conventional TUR, blood transfusion, or additional surgical procedures, in this study. Thus, we emphasize that our EMR + en-bloc method has some advantages for resection of relatively larger tumors.

A randomized study of 142 patients showed that there was no significant difference in recurrence rates achieved with en-bloc resection and conventional TUR (*p* = 0.383) [20]. In addition, a multicenter European study of 221 patients, supported this finding [17]. Although our study population is relatively small as compared to those in other studies, we also found a comparable rate of recurrence between patients in the EMR + en-bloc resection and the conventional TUR groups. Furthermore, a previous report showed that the recurrence rates after 12 months were 24.5 and 18.5% for E-EBRT and laser en-bloc resection of the tumor (L-EBRT), respectively [17]. Comparatively, in our study, the recurrence rate observed in the EMR + en-bloc resection group was 15.4% after 12 months. Thus, the recurrence rate of BC observed with our en-bloc resection method was similar to, or even better than that achieved with the L-EBRT technique.

The major limitations of this study include its non-randomized design and the relatively small study-population (which also affected collection of follow-up data). However, we believe that our findings are significant as those of a preliminary study, because this is the first report of the utilization of an original, easily adopted, and cost-effective EMR + en-bloc resection technique that may be used effectively in patients with NMIBC. In addition, this operation was performed by a single surgeon in a single institution, and clinicopathological features were matched between the EMR + en-bloc and TUR groups. Therefore, biases occurring due to surgical technique and patient background were kept to a minimum. However, another limitation was that the pathologists could not be blinded, as they were able to determine the surgical method employed, from the histopathological characteristics of resected tissues. However, this was a retrospective study and the two pathologists were not made aware of the design and significance of the study when they made the histopathological diagnosis. Furthermore, another limitation is that the health insurance system in Japan differs from those in other countries. Consequently, certain data, including duration of catheterization, were influenced because their costs were covered by this system, which should be considered during data analysis. However, we believe that the influence of such differences is not that significant on our discussion because they were comparable across both treatment groups. Finally, we recommend that further detailed, large-scale research based on the results of this preliminary study are necessary to determine the safety and usefulness of the EMR + en-bloc resection technique in patients with BC. Long-term clinical studies with inclusion of patients with larger-sized tumors are important to determine and improve upon the efficacy and safety of this original operative approach.

## Conclusions

Our results showed that the novel EMR + en-bloc resection technique is feasible, useful, and safe for treating patients with NMIBC. In addition, an accurate pathological diagnosis can be reached, using this technique. Further large-scale, multicenter, randomized controlled trials with long-term follow-up, are needed to validate our findings and to improve the long-term outcomes in patients with NMIBC.

## Data Availability

All the study data are included in this manuscript.

## Abbreviations

BC: Bladder cancer
E-EBRT: Electrical en-bloc resection of the tumor
EMR: Endoscopic mucosal resection
NMIBC: Non-muscle invasive bladder cancer
TUR: Transurethral resection
